# Prevalence and characteristics of family history of sudden unexplained death and predictors of negative attitude of family members toward medical autopsy and family screening in Saudi Arabia: A cross-sectional study

**DOI:** 10.1371/journal.pone.0277914

**Published:** 2022-11-23

**Authors:** Wael Alqarawi, Nouf Bin Muammar, Nuha Alajlan, Tarek Kashour, Ahmad Hersi

**Affiliations:** 1 Department of Cardiac Sciences, College of Medicine, King Saud University, Riyadh, Saudi Arabia; 2 University of Ottawa Heart Institute, University of Ottawa, Ottawa, Canada; 3 Department of Emergency Medicine, Ad Diriyah Hospital, Ministry of Health, Riyadh, Saudi Arabia; 4 Department of Internal Medicine, King Abdulaziz Medical City, Riyadh, Saudi Arabia; Prince Sattam bin Abdulaziz University Faculty of Medicine, SAUDI ARABIA

## Abstract

**Introduction:**

Little is known about sudden unexplained death (SUD) in Saudi Arabia. Moreover, family screening and medical autopsy are not routinely performed due to perceived religious and cultural resistance. However, this has never been systematically examined. We sought to describe the prevalence and characteristics of family history of SUD and the attitude of family members toward medical autopsy and family screening.

**Methods:**

This was a cross-sectional study utilizing an online survey distributed though social media platforms from August 15 to September 15, 2021. Participants’ characteristics, details about SUD cases, and the attitude toward medical autopsy and family screening were collected. Multivariable logistic regression was used to identify independent predictors of negative attitude toward medical autopsy.

**Results:**

A total of 11374 were included in the final analysis after excluding children. The prevalence of FHx of at least one first degree relative (FDR) with SUD was found to be 9.4% [95% CI (8.9% - 10%)]. Among participants with any FHx of SUD, 1346/3489 (38.6%) had ≥ 2 family members affected. Only 183 participants with a FHx of SUD visited a physician for the purpose of family screening (183/3489, 5.3%). The total number of SUD cases reported was 5474. Of those, 22% were 35-year-old or younger. Only 22% of participants (2458/11374) had a negative attitude towards medical autopsy, and the most common reason was the perceived lack of benefit. Older age (> 35 years), family history of SUD, female gender, and lack of knowledge about the yield of medical autopsy were associated with negative attitude in the adjusted analysis.

**Conclusion:**

SUD occurred at young age and affected multiple family members in a significant proportion of families. Despite that, family screening was seldom performed. There is an urgent need to improve the care of SUD by incorporating medical autopsy and developing clinical pathways to screen family members.

## Introduction

Sudden cardiac death (SCD) refers to sudden and unexpected death from a presumed cardiac cause in a person with or without an underlying cardiac disease [1]. When the cause of SCD is not known, the term sudden unexplained death (SUD) is used (either because no autopsy was performed or because autopsy did not reveal a cause) [1]. Previous studies have established the critical role of medical autopsy and family screening in identifying the cause of SUD in order to prevent recurrent events in the family, and these have been endorsed by scientific societies [2–6]. However, there is limited data on the uptake of these recommendations in Saudi Arabia. Moreover, the characteristics of SUD and the care provided to their family members are essentially unknown. The Saudi population has a unique age distribution that might affect the prevalence and causes of SUD and religious and cultural practices that influence the care provided to families with a history of SUD. As such, we conducted this study to describe the prevalence and characteristics of SUD, the care provided to family members and their attitudes toward screening and medical autopsy.

## Method

This was a cross-sectional study, utilizing anonymous online survey distributed through social media platforms (i.e. Twitter, WhatsApp). A structured questionnaire was developed using the Google tool (Google Forms). It was developed in English and then translated into Arabic by the authors. SUD was defined as sudden, unexpected and unexplained death within 24 hours of last being observed in normal health and family history of SUD was defined as having any first or second degree relative who had SUD. Medical terms [SUD and first degree relatives (FDR)] were explained in details at the beginning of the survey. The Arabic version was piloted on 100 individuals and a proportion of them were contacted to ensure proper understanding of the terminology used and to adjust answer options to capture their responses more precisely. The initial version of the survey was then revised, and results of the pilot sample were not included in the analysis. The final version of the survey was conducted from August 15 to September 15, 2021 and included only adults (≥ 18 years old). Highly influential social media users with diverse followers were approached to distribute the survey.

The survey included questions regarding the demographics of participants, characteristics of family history of SUD, and knowledge and attitudes toward medical autopsy and family screening of individuals with family history of SUD. All were multiple choice questions to ensure complete data capture except for one question that had an option for free text (the possible cause of death). This was done to reduce inclusion of SUD cases where a potential explanation is known (e.g. “SUD” due to known congenital heart disease or known coronary artery disease). Full list of questions and answer options can be found in S1 Fig in S1 File.

### Ethical considerations

An informed written consent was obtained from patients at the beginning of the survey. The study was reviewed and approved by the King Saud University College of Medicine institutional review board.

### Statistical analysis

Continuous data were reported as mean (standard deviation) and categorical variables were summarized as percentages. Chi Square test, Fisher’s exact test and Student’s t-test were used as appropriate. Age of deceased was categorized into young adults versus older adults, using 35 years as a cut-off. This was based on previous literature which showed a distinctly higher prevalence of inherited cardiac conditions (ICC) as the cause of SUD in young adults [7–10]. A pre-specified sensitivity analysis excluding participants where the cause of “SUD” could have been explained or not fulfilling the definition of SUD was performed.

We used multivariable logistic regression modeling to identify independent predictors of negative attitude toward medical autopsy. The following variables were included in the model: family history of SUD, previous knowledge that autopsy can reveal the cause of SUD, age of participant, level of education, gender, nationality, and occupation (physician). Age was categorized into young adults versus older adults and level of education was categorized into bachelor’s or higher degrees versus lower degrees. Analyses were performed using SAS (version *9*.*4*, The *SAS* institute, USA) and p values of < 0.05 were considered statistically significant.

## Results

### Participants’ characteristics

**Fig 1:**
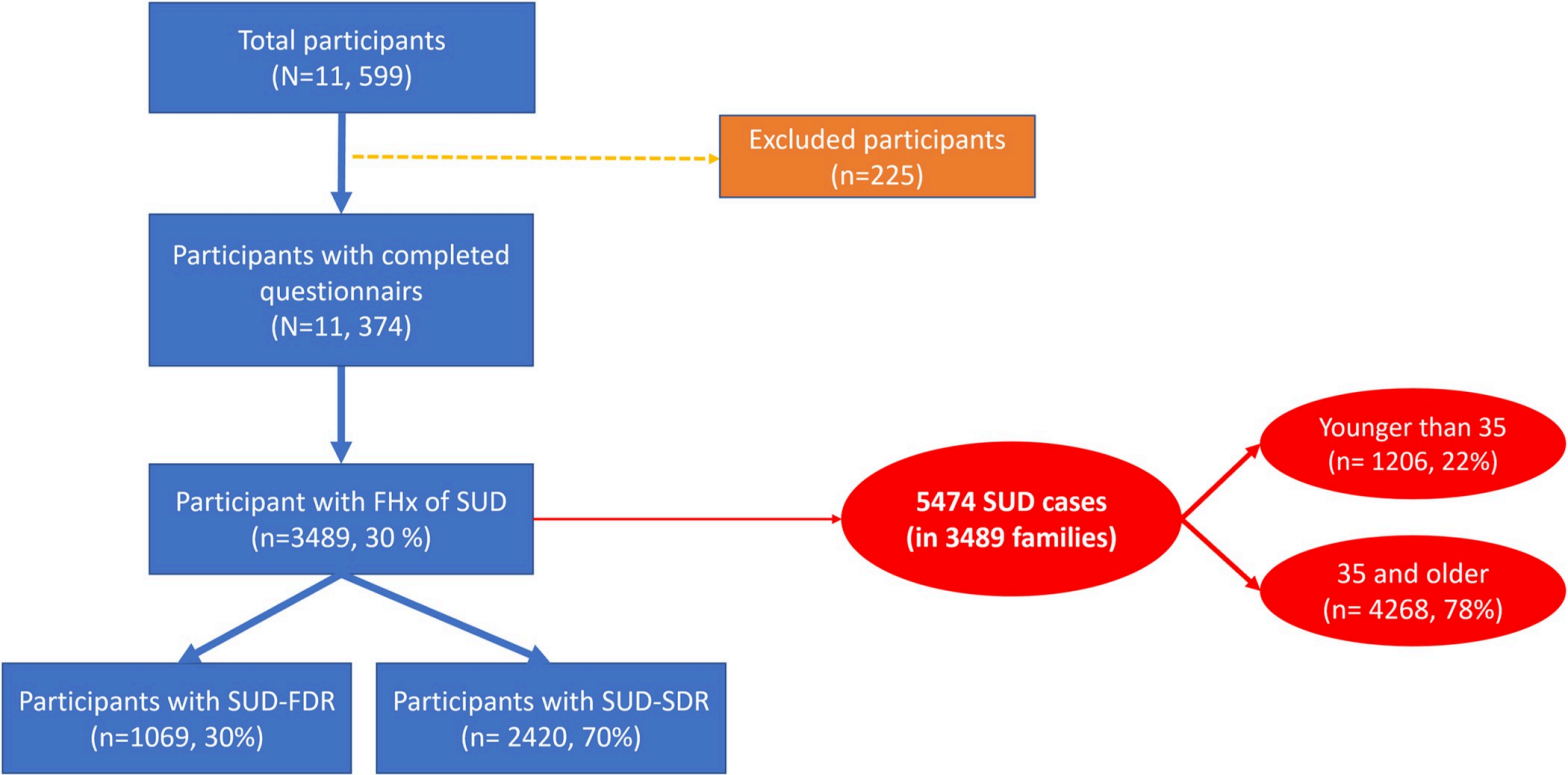
Study flowchart. Study flowchart depicting the prevalence of family history of sudden unexplained death and the number of sudden explain death cases. FHx: family history, SUD: sudden unexplained death, SUD-FDR: sudden unexplained death in at least one first degree relative, SUD-SDR: sudden unexplained death in second degree relative(s).

Fig 1 shows the study flowchart. A total of 11,599 participants agreed to participate, of which 11374 were included in the final analysis after excluding children (≤ 18 years old). Most participants were 35-year-old or younger (6849/11374, 60.2%) and 70.4% were male gender (8004/11374). All geographical regions of Saudi Arabia were represented, with Riyadh region being the most common residence (5971/11374, 52.5%). Only 1.8% of the responders were physicians (206/11374). Table 1 summarizes the characteristics of participants.

**Table 1:** Characteristics of participants.

Variable	Category	No. (%) of responders
Gender	Male	8004 (70.37%)
	Female	3370 (26.63%)
Age (Year)	18–25	2794 (24.56%)
	26–35	4055 (35.65%)
	36–50	3603 (31.67%)
	51–65	838 (7.36%)
	>65	84 (0.73%)
Nationality	Saudi	10883 (95.68%)
	Non-Saudi	491 (4.32%)
Region	Riyadh	5971 (52.5%)
	Al-Qaseem	830 (7.30%)
	Makkah	1631 (14.34%)
	Madinah	481 (4.23%)
	Eastern	1265 (11.12%)
	Tabuk	207 (1.82%)
	Al-Jawf	75 (0.66%)
	Northern	85 (0.75%)
	Hail	147 (1.29%)
	Najran	76 (0.67%)
	Jazan	149 (1.31%)
	Aseer	372 (3.27%)
	Al-Bahah	85 (0.75%)
Level of education	Primary school	39 (0.34%)
	Secondary school	150 (1.32%)
	High school	2213 (19.46%)
	Bachelor degree	7477 (65.74%)
	Postgraduate	1495 (13.14%)
Occupation	Physician	206 (1.81%)
	Non-physician	11168 (98.19)

### Prevalence of family history of SUD

A total of 3489 participants had FHx of SUD (3489/11374, 30.7%). Of those, 1069 (1069/3489, 30.6%) had at least one FDR with SUD (SUD-FDR). Thus, the prevalence of FHx of at least one FDR with SUD was found to be 1069/11347 (9.4%) [95% CI (8.9% - 10%)].

### Characteristics of family history of SUD

Table 2 summarizes characteristics of FHx of SUD, comparing those with at least one FDR (SUD-FDR) versus those with only second degree relative (SUD-SDR). Among participants with any FHx of SUD, 1346/3489 (38.6%) had ≥ 2 family members affected. Only 183 participants with FHx of SUD visited a physician for the purpose of family screening (183/3489, 5.3%), which was more common in the SUD-FDR group as compared to the SUD-SDR group (9.1% vs 3.6%, p<0.0001). ICCs were perceived as possible causes of SUD in only 4.2% of the cases.

**Table 2:** Characteristics of family history of sudden unexplained death (SUD).

Variable	Total (3489)	SUD-FDR (1069)	SUD-SDR (2420)	P value
Number of SUD cases per family	1.54 (0.85)	1.64 (0.97)	1.54 (0.83)	0.002
Number of SUD cases per family				0.005
One	2143 (61.4%)	639 (59.8%)	1504 (62.2%)	
Two	922 (26.4%)	272 (25.4%)	650 (26.9%)	
Three	281 (8.1%)	96 (9.0%)	185 (7.6%)	
Four	71 (2.0%)	28 (2.6%)	43 (1.8%)	
Five	72 (2.1%)	34 (3.2%)	38 (1.6%)	
≥2 family member affected	1346 (38.6%)	430 (40.2%)	916 (37.9%)	0.184
At least one SUD at young age*	844 (24.2%)	249 (22.9%)	599 (24.8%)	0.244
All SUD cases at young age	544 (15.6%)	145 (13.6%)	399 (11.4%)	0.028
Perceived possible cause of death				<0.001
Unknown	2453 (70.3%)	705 (65.9%)	1747 (72.2%)	
CAD	510 (14.6%)	172 (16.1%)	338 (14%)	
Inherited cardiac condition	147 (4.2%)	47 (4.4%)	100 (4.1%)	
Others	380 (10.9%)	145 (13.6%)	235 (9.7%)	
Family screening performed (yes)	183 (5.3%)	97 (9.1%)	86 (3.6%)	<0.001

SUD-FDR: Participants with at least one first degree relative with SUD, SUD-SDR: participants with second degree relative(s) with SUD, CAD: coronary artery disease. * young age is defined as 35 years or younger.

The total number of SUD cases reported was 5474. Of those, 78.7% were males (3585/4555) and 22% were young adults (1206/5474).

### Attitudes toward medical autopsy and family screening

**Fig 2:**
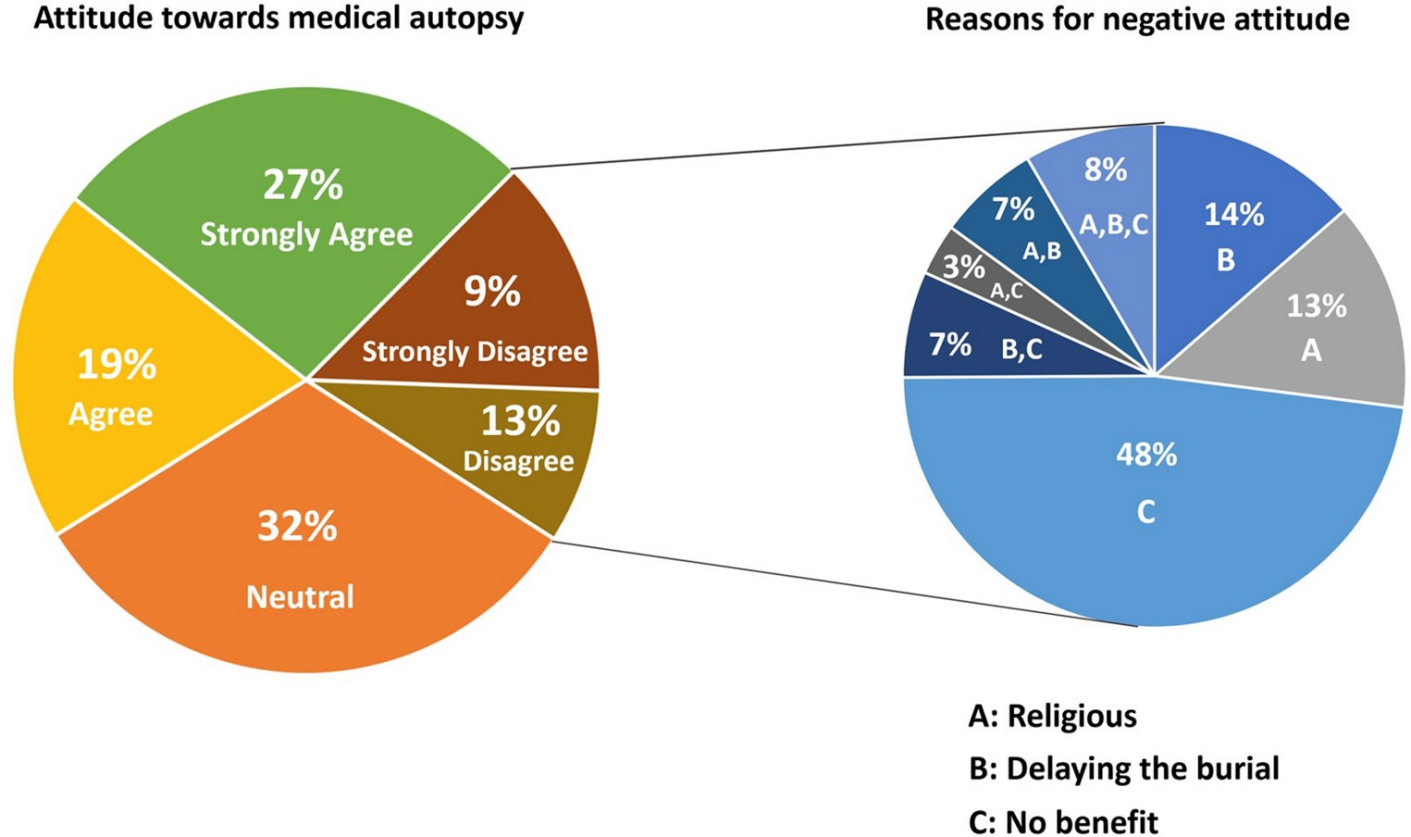
Attitude towards medical autopsy and reasons for negative attitude. Attitude of participants toward medical autopsy and reasons for negative attitude (disagree/strongly disagree).

Seventy three percent of participants believed that FDRs of SUD should be examined by a physician, (8306/11374, 73%). Fig 2 shows the attitude of participants toward medical autopsy and reasons for negative attitude (disagree/strongly disagree). In those who knew that medical autopsy can reveal the cause of SUD, negative attitude was significantly lower than those who did not know [1164/6066 (19.2%) vs 1294/5308 (24.4%), p<0.001]. Predictors of negative attitude are shown in Table 3. Older age (> 35 years), family history of SUD, female gender and lack of knowledge about the yield of medical autopsy were associated with negative attitude in the adjusted analysis. The most common reason for the negative attitude was the perceived lack of benefit from medical autopsy.

**Table 3:** Univariable and multivariable analysis of potential predictors of negative attitude toward medical autopsy.

Variable		Univariable analysis			Multivariable analysis	
	OR	95% CI	P	OR	95% CI	P
Family history of SUD	**1.314**	**1.196–1.444**	**<0.001**	**1.305**	**1.186–1.436**	**<0.001**
Age of participant (> 35 years)	**1.727**	**1.578–1.890**	**<0.001**	**1.750**	**1.598–1.916**	**<0.001**
Level of education (≥ bachelor’s)	1.054	0.945–1.174	0.345	1.044	0.935–1.166	0.445
Nationality (Saudi)	1.024	0.823–1.274	0.831	1.037	0.831–1.294	0.750
Gender (Female)	1.092	0.991–1.203	0.075	**1.190**	**1.077–1.314**	**<0.001**
Occupation (physician)	0.739	0.512–1.066	0.105	0.847	0.584–1.228	0.3815
Previous knowledge about the yield of medical autopsy (yes)	**1.358**	**1.241–1.485**	**<0.001**	**1.371**	**1.251–1.502**	**<0.001**

SUD: sudden unexplained death.

### Sensitivity analysis

Out of the 3489 participants with a FHx of SUD, 338 were excluded in this pre-specified sensitivity analysis due to a possible cause of death (338/3489, 9.7%). The most common reasons for exclusions are listed in S1 Table in S1 File. S2 Table in S1 File shows the characteristics of FHx of SUD which is similar to the main analysis, with the exception of a higher prevalence of SUD at young age especially in the SUD-SDR group. Independent predictors of a negative attitude toward medical autopsy were similar to the main analysis as shown in S3 Table in S1 File.

## Discussion

Our study reports for the first time the prevalence and characteristics of family history of SUD in Saudi Arabia, and the attitude of family members toward medical autopsy and family screening. Three major findings were revealed in this cross-sectional study. First, 22% of SUD victims were younger than 35-year-old. Second, less than 10% FDRs of SUD victims were screened. Third, only 22% of participants had a negative attitude toward medical autopsy, and the most common reason was the perceived lack of benefit. These findings have important clinical and research implications that will hopefully help improve the care of SUD in Saudi Arabia.

The age of SUD is an important factor that determine the way we approach family members. Indeed, the recent guidelines suggest that genetic testing for family members with no phenotype is only reasonable when the deceased is young. This is because ICCs are the main causes of SUD at young age, as compared to older adults where the vast majority are due to undiagnosed CAD [9–11]. While all SUDs are tragic, SUD at young age is more devastating due to the implications on family members and the years of potential life lost. Our finding that 22% of SUD reported where at young age should ignite the interest in improving the care of SUD in Saudi Arabia by developing clinical pathways to screen family members.

Despite the clear recommendation to screen FDRs of SUD victims, we found that only 9.4% were screened in the current study [12]. The fact that 40% of SUD-FDR families have more than one SUD case highlight the lost opportunity of potentially preventing subsequent cases had a proper evaluation been performed after the first SUD case. This is particularly troublesome because a large proportion of SUD happened at young age, where the cause of SUD is more likely to be an ICC [9–11]. In addition, medical autopsy is seldom performed in Saudi Arabia, which renders family screening the only way to identify inherited conditions and potentially prevent further tragedies. When one examines the causes of SUD at young age in general, 2 important messages relevant to family screening need to be discussed. First, SCD can be the presenting symptom of most of these conditions, highlighting the importance of screening as opposed to waiting for symptoms to develop. Indeed, Long QT Syndrome (LQTS), Catecholaminergic Polymorphic Ventricular Tachycardia (CPVT) and Arrhythmogenic Right Ventricular Cardiomyopathy (ARVC) are amongst the most common conditions revealed by family screening and are known for presenting with fetal arrhythmias at initial presentation [4, 13]. Second, effective therapies that are shown to reduce the incidence of SCD in these conditions have been well established such as Beta-Blockers for LQTS and CPVT and ICD for ARVC. While the yield of family screening in identifying the cause of SUD has been shown to be modest in previous studies (10–22%), one needs to consider that these studies included mainly families where the autopsy failed to reveal the cause of SUD [4, 6, 14, 15]. As such, it is reasonable to postulate that the yield might be different when autopsy is systematically not performed (like the practice in Saudi Arabia), and one needs to study this in order to define the yield of family screening.

Medical autopsy is the cornerstone of evaluating SUD as it reveals the cause in the majority of cases [2, 3, 5, 9]. This helps guide family screening when the cause is an ICC and alleviate anxiety and avoid unnecessary investigations when the cause is found be an acquired disease. Unfortunately, however, there is no system for medical autopsy in Saudi Arabia [16]. This is thought to be due to religious and cultural challenges. However, our study challenges this perception as it showed that only 22% of participants had a negative attitude toward medical autopsy, with the most common reason being the perceived lack of benefit from autopsy. Not surprisingly, we found that lack of knowledge about the yield of autopsy was independently associated with negative attitude. Nonetheless, even in those who knew that autopsy can reveal the cause of SUD, lack of benefit remained the most common reason for the negative attitude. It is likely that this can be explained by the limited public awareness that ICCs are the main causes of SUD, especially at young age. This is supported by our finding that only 4% of family members of SUD thought that ICC is the possible cause of SUD. In other words, these participants believed that even if the autopsy reveal the cause of SUD, this will not affect their risk of SCD because ICC is not on their mind as a possible cause of SUD. We believe that this should guide our efforts toward improving public awareness not only on the yield of autopsy but also the importance of ICCs in SUD cases.

Our study has important limitations. First, we relied on social media users who do not necessarily represent the whole Saudi population. There is certainly a selection bias when using social medial platforms where certain populations are less likely to be reached such as older individuals, rural populations and those with low socioeconomic status. Nevertheless, according to the latest statistics, 80% of Saudis are active users of social medial platforms and, as such, the study population represents the majority of the Saudi population [17]. Separately, we collected self-reported SUD, which can be inaccurate. This is especially important because the terms used in this study such as SUD and SCD can be confusing. Nonetheless, we implemented several steps to mitigate that risk. First, we piloted our questionnaire on 100 participants and revised our language to ensure proper understanding of questions. Second, we explained important terminology at the beginning of the questionnaire using simple and non-medical language. Last, we performed a sensitivity analysis excluding cases where it was potentially not fulfilling our SUD definition. lastly, our study was a cross-sectional survey using a non-probability sampling and, as such, has the inherit limitations of that technique.

## Conclusion

The care provided to Saudi families with SUD is suboptimal. There is a need to establish clinical pathways to screen FDRs and to perform medical autopsy in SUD. Public campaigns to increase awareness about the importance of ICCs in SUD can potentially improve the already acceptable attitude toward medical autopsy.

## Supporting information

S1 File (DOCX)
