# Supplementary material for: Prevalence and characteristics of family history of sudden unexplained death and predictors of negative attitude of family members toward medical autopsy and family screening in Saudi Arabia: A cross-sectional study
Source: PLoS One. 2022 Nov 23;17(11):e0277914. doi: 10.1371/journal.pone.0277914 (PMC9683581; doi:10.1371/journal.pone.0277914)
Supplement: S1 File — (DOCX) [file pone.0277914.s001.docx]

Table1S: Common examples for exclusion in the sensitivity analysis:

| Intracranial hemorrhage |
| --- |
| Known to have a SCD-predisposing condition such as coronary artery disease |
| Car accident |
| Seizure |
| Medication side effects |
| Coronavirus disease-19 (COVID-19) |
| Hypoglycemic episode |
| Chocking |

SCD: sudden cardiac death

Table2S: Characteristics of family history of sudden unexplained death (SUD):

| Variable | Total (3151) | SUD-FDR (915) | SUD-SDR (2236) | P value |
| --- | --- | --- | --- | --- |
| Number of SUD cases per family | 1.56 (0.87) | 1.53 (0.82) | 1.57 (0.89) | <0.001 |
| Number of SUD cases per family  One  Two  Three  Four  Five | 1949 (61.9%)  826 (26.2%)  253 (8%)  64 (2%)  59 (1.9%) | 573 (62.2%)  234 (25.6)  77 (8.4%)  24 (2.6%)  7 (0.8%) | 1376 (61.5%)  592 (26.5%)  176 (7.9%)  40 (1.8%)  52 (2.3%) | 0.024 |
| >2 family member affected | 1202 (38.2%) | 342 (37.4%) | 860 (38.7%) | 0.569 |
| At least one SUD at young age* | 796 (25.3%) | 196 (21.4%) | 600 (26.8%) | 0.002 |
| All SUD cases at young age | 585 (18.6%) | 136 (14.9%) | 449 (20.1%) | <0.001 |
| Perceived possible cause of death  Unknown  CAD  Inherited cardiac condition  Others | 2452 (77.8%)  510 (16.2%)  147 (4.7%)  380 (12.1%) | 705 (65.9%)  172 (16.1%)  47 (4.4%)  145 (13.6%) | 1747 (72.2%)  338 (14%)  100 (4.1%)  235 (9.7%) | <0.001 |
| Family screening performed (yes) | 157 (5%) | 79 (8.6%) | 78 (3.5%) | <0.001 |

SUD-FDR: Participants with at least one first degree relative with SUD, SUD-SDR: participants with second degree relative(s) with SUD, CAD: coronary artery disease. * young age is defined as 35 years or younger.

Table3S: Univariable and multivariable analysis of potential predictors of negative attitude toward medical autopsy:

| Variable |  | Univariable analysis |  |  | Multivariable analysis |  |
| --- | --- | --- | --- | --- | --- | --- |
|  | OR | 95% CI | P | OR | 95% CI | P |
| Family history of SUD | **1.314** | **1.196 – 1.444** | **<0.001** | **1.305** | **1.186 – 1.436** | **<0.001** |
| Age of participant (> 35 years) | **1.727** | **1.578 – 1.890** | **<0.001** | **1.750** | **1.598 – 1.916** | **<0.001** |
| Level of education (> bachelor's) | 1.054 | 0.945 – 1.174 | 0.345 | 1.044 | 0.935 – 1.166 | 0.445 |
| Nationality (Saudi) | 1.024 | 0.823 – 1.274 | 0.831 | 1.037 | 0.831 – 1.294 | 0.750 |
| Gender (Female) | 1.092 | 0.991 – 1.203 | 0.075 | **1.190** | **1.077 – 1.314** | **<0.001** |
| Occupation (physician) | 0.739 | 0.512 – 1.066 | 0.105 | 0.847 | 0.584 – 1.228 | 0.3815 |
| Did not know that medical autopsy can reveal the cause of SUD | **1.358** | **1.241 – 1.485** | **<0.001** | **1.371** | **1.251 – 1.502** | **<0.001** |

SUD: sudden unexplained death.


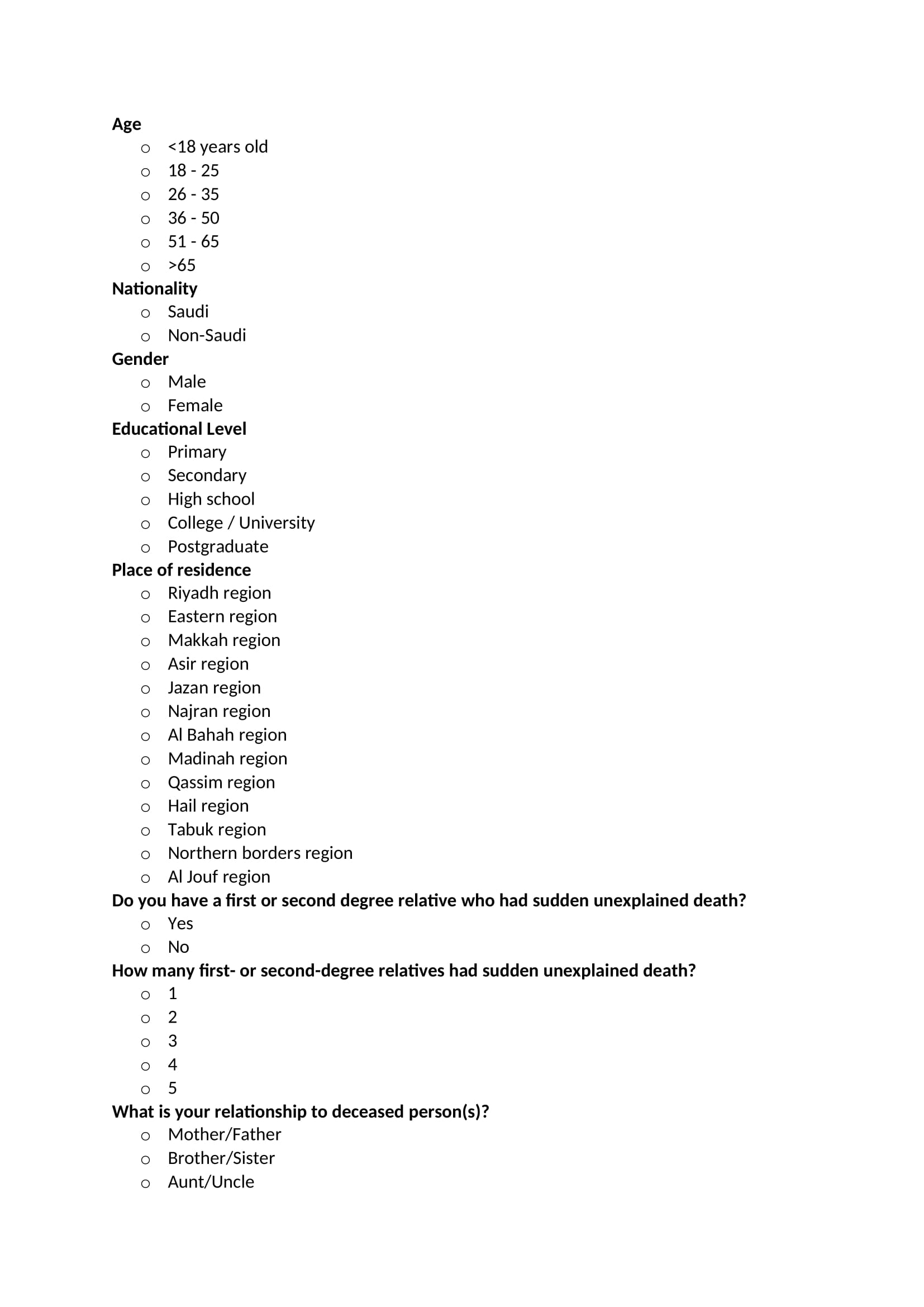


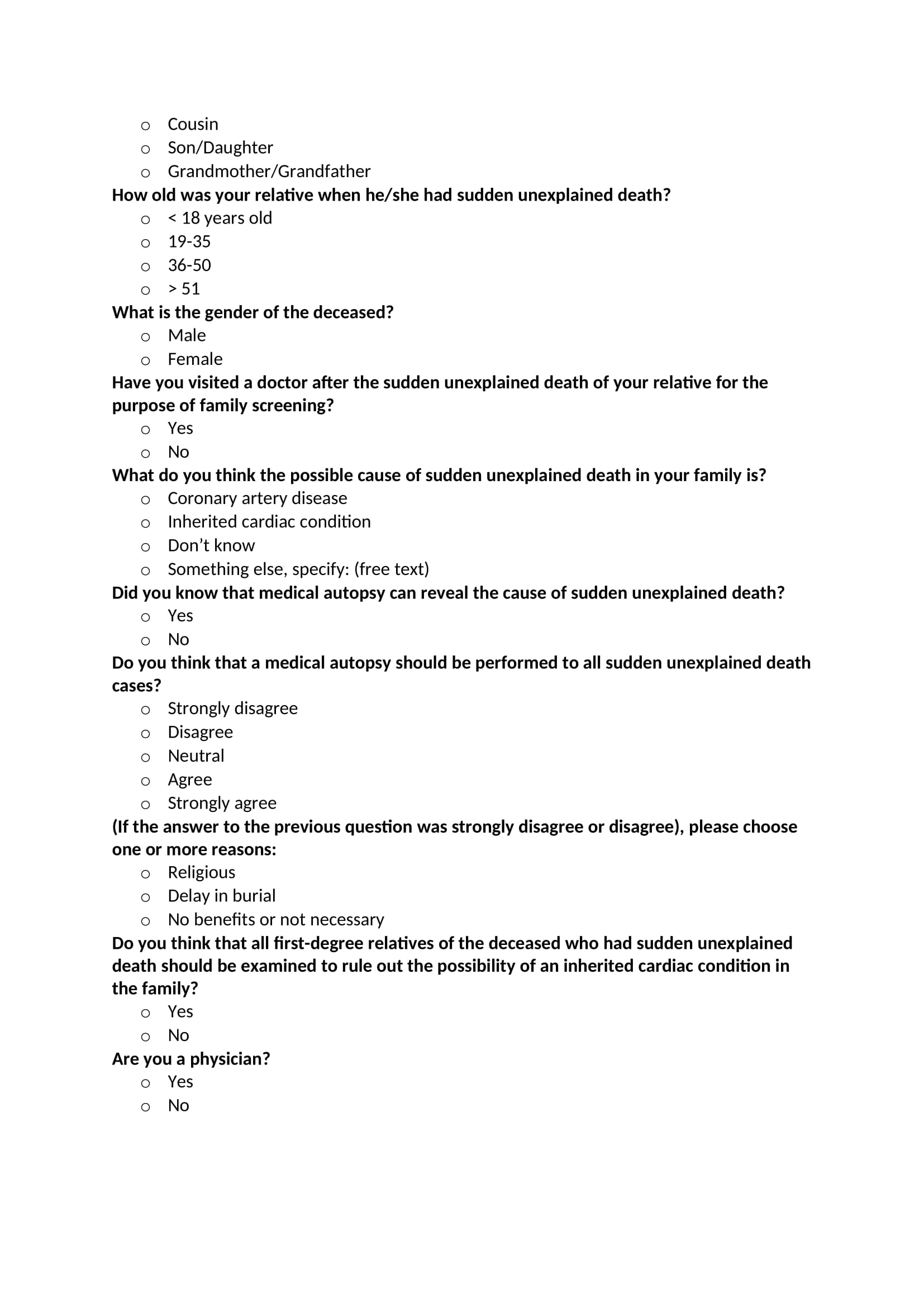


Figure 1S: The list of questions and answer options used in the inline survey
