# Supplementary material for: Prevalence and characteristics of family history of sudden unexplained death and predictors of negative attitude of family members toward medical autopsy and family screening in Saudi Arabia: A cross-sectional study
Source: PLoS One. 2022 Nov 23;17(11):e0277914. doi: 10.1371/journal.pone.0277914 (PMC9683581; doi:10.1371/journal.pone.0277914)
Supplement: Attachment — Submitted filename: Response to Reviewers.docx [file pone.0277914.s002.docx]

Oct 27, 2022

**RE:** “The Prevalence and Characteristics of Family History of Sudden Unexplained Death and The Attitude of Family Members Toward Medical Autopsy and Family Screening: A Cross-Sectional Study with 11,000 Participants in Saudi Arabia” (PONE-D-22-22713)

Dear Dr. Chenette,

The authors would like to thank the editor and reviewers for taking the time to appraise our manuscript. We have revised the manuscript based on the reviewers’ suggestions. The comments are in bold and our responses follow.

Reviewer #1

**Even though the study have major limitations in its design, this study represents very important data and new knowledge deriving from a part of the world that do not represent the data we usually refer to when discussing SCD. The authors are to be commended for this!**

Thank you. Indeed, we hope that this manuscript will raise awareness and inform decisions about the care of SCD not only in Saudi Arabia but also in other countries that share similar cultural and religious backgrounds.

**I would moderate the conclusion in abstract. You state “SUD occurred at young age and affected multiple family members in a significant proportion of families. Despite that, family screening was seldom performed.” This is not wrong, but you have to keep in mind, that the sample you are describing do not represent the general population in Saudi. This is a Social Media questionnaire that only reached some parts of the community (the young?), and maybe only were returned by those severely affected by the questions? Nonetheless, the data are very disturbing and should warrant further investigations and information campaigns et cetera**

**Limitations:
Bias on collection through SoMe of course makes it impossible to say that this cohort represents the general population. They are most likely younger and more urban?
Who answers these questionnaires? And more important – who wont receive the questionnaire?
This should be discussed too**

Thank you for allowing us to clarify this important point. We completely agree with the reviewer that selection bias is an important limitation of our study which might have affected the characteristics of responders. We have revised our conclusion in the abstract and revised our limitations to discuss selection bias.

- Line 62: Revised “SUD occurred at young age and affected multiple family members in a significant proportion of responders”
- Line 241: Added “There is certainly a selection bias when using social medial platforms where certain populations are less likely to be reached such as older individuals, rural populations and those with low socioeconomic status.”

**Line 216-218: The yield in family screening in families with structural heart disease is higher, and in SADS (negative autopsy) around 20%. The yield is lower in non-autopsied families, presumably because not all SD are in fact SCD (and not all SCD are due to inherited cardiac disease). Please revise**

Thank you for your thoughtful comment. We have revised the manuscript.

- Line 218: Revised “As such, it is reasonable to postulate that the yield might be different when autopsy is systematically not performed (like the practice in Saudi), and one needs to study this in order to define the yield of family screening.

**On a smaller note, I would keep SUD for those that are unexplained, either because no autopsy was performed or because autopsy did not reveal a cause. I would use the term SD (sudden death) as a collection of these entities, which could then be divided in non-autopsied SD (which could then be SUD or SCD pr definition), and autopsied SD, which could then be non-cardiac SD, SCD or SUD/SADS. This would also be in line with current guidelines

I think this paper is very important, politically it should be used to affect policy makers, and to design campaigns towards the population to raise awareness on this important topic**

We have revised our manuscript to clarify the definition of SUD, as suggested.

- Line 70: Added “(either because no autopsy was performed or because autopsy did not reveal a cause)”.

Reviewer #2

**Saudi within the title and manuscript should be replace by Saudi Arabia.**

“Saudi” was replaced by “Saudi Arabia” throughout the manuscript.

**I believe the title should be a bit edited by deleting the number of participants and adding independent predictors of negative attitude toward medical autopsy. therefore, I suggest the title to be: Prevalence, Characteristics of Family History of Sudden Unexplained Death and Predictors of Negative Attitude of Family Members Toward Medical Autopsy and Family Screening in Saudi Arabia: A Cross-Sectional Study.**

We have revised both the long and short titles as suggested.

- Line 1: Revised “Prevalence and Characteristics of Family History of Sudden Unexplained Death and Predictors of Negative Attitude of Family Members Toward Medical Autopsy and Family Screening in Saudi Arabia: A Cross-Sectional Study.”
- Line 6: Revised “Family History of Sudden Unexplained Death in Saudi Arabia: A Cross-Sectional Study”

**The objective should be strictly aligned with the title. I do not see prevalence in the objective statement.**

Thank you for highlighting this important point. We have revised the manuscript to add this objective.

- Line 42: Added “We sought to describe the prevalence and characteristics of family history of SUD and the attitude of family members toward medical autopsy and family screening”.
- Line 81: Added “As such, we conducted this study to describe the prevalence and characteristics of SUD, the care provided to family members and their attitudes toward screening and medical autopsy.”

**FHx should be defined.**

Manuscript was revised to define FHx.

- Line 89: Added “and family history of SUD was defined as having any first or second degree relative who had SUD.”

**"The Saudi population has a unique age distribution and religious and cultural practices that might affect the prevalence and causes of SUD and care provided to their families". This sentence should be rewritten to add clarity and avoid any confusion in regard of age and practices with causes and care.**

The sentence was revised.

- Line 79: Revised “The Saudi population has a unique age distribution that might affect the prevalence and causes of SUD and religious and cultural practices that influence the care provided to families with a history of SUD.”

**Details about questionnaire development, validation and reliability should be provided in the methods section.**

Thank you for your suggestion. We added a sub-heading in the method section detailing our questionnaire’s development and content and discussed the assessment of validity and reliability of the questionnaire. We also added to our discussion a limitation of not calculating any formal statistics related to reliability and validity, although we contacted responders during the pilot phase to ensure these qualities.

- Line 86: Added a subheading “*Questionnaire development and content”*
- Line 259: Added “Third, although we contacted responders to ensure reliability and validity of the questionnaire, we did not calculate any formal statistics to objectively report that.”

**the prevalence was not clearly given, I believe it is 5474? or 3489?!. I believe a better presentation of outputs statring from larger to smaller should be presented clearly.**

Thank you for allowing us to clarify this important point. The number of participants with a family history of SUD was 3489 (3489/11374, 30.7%). However, some of them had more than one family member with SUD and, as such, the total number of SUD *cases* was higher (5474 SUD cases). We have updated our manuscript with a figure that explains these numbers.

- Added the following figure:

Fig 1: Study flowchart depicting the prevalence of family history of sudden unexplained death and the number of sudden explain death cases. FHx: family history, SUD: sudden unexplained death, SUD-FDR: sudden unexplained death in at least one first degree relative, SUD-SDR: sudden unexplained death in second degree relative(s).

**limit geographical location to cities rather than cities and regions( Eastern and Northern to be replaced by corresponding cities)**

All geographical locations are regions. Some regions are named after the main city (e.g. Riyadh region, Mecca region) and others were named according to their location (e.g. Eastern region, Northern region). We added the word “region” to their names to avoid any confusion

- Table 1. Added “region” to all regions.
