# Supplementary material for: Prevalence and characteristics of family history of sudden unexplained death and predictors of negative attitude of family members toward medical autopsy and family screening in Saudi Arabia: A cross-sectional study
Source: PLoS One. 2022 Nov 23;17(11):e0277914. doi: 10.1371/journal.pone.0277914 (PMC9683581; doi:10.1371/journal.pone.0277914)
Supplement: Peer review history

# Peer review: Decision Letter 0

**DOI:** 10.1371/journal.pone.0277914.r001

**Authors:** Mohammed H. Karrar Alsharif

25 Oct 2022

PONE-D-22-22713The Prevalence and Characteristics of Family History of Sudden Unexplained Death and The Attitude of Family Members Toward Medical Autopsy and Family Screening: A Cross-Sectional Study with 11,000 Participants in Saudi ArabiaPLOS ONE

Dear Dr. alqarawi,

Thank you for submitting your manuscript to PLOS ONE. After careful consideration, we feel that it has merit but does not fully meet PLOS ONE’s publication criteria as it currently stands. Therefore, we invite you to submit a revised version of the manuscript that addresses the points raised during the review process.

Please submit your revised manuscript by Dec 09 2022 11:59PM. If you will need more time than this to complete your revisions, please reply to this message or contact the journal office at plosone@plos.org. When you're ready to submit your revision, log on to https://www.editorialmanager.com/pone/ and select the 'Submissions Needing Revision' folder to locate your manuscript file.

Please include the following items when submitting your revised manuscript:

- A rebuttal letter that responds to each point raised by the academic editor and reviewer(s). You should upload this letter as a separate file labeled 'Response to Reviewers'.
- A marked-up copy of your manuscript that highlights changes made to the original version. You should upload this as a separate file labeled 'Revised Manuscript with Track Changes'.
- An unmarked version of your revised paper without tracked changes. You should upload this as a separate file labeled 'Manuscript'.

If you would like to make changes to your financial disclosure, please include your updated statement in your cover letter. Guidelines for resubmitting your figure files are available below the reviewer comments at the end of this letter.

If applicable, we recommend that you deposit your laboratory protocols in protocols.io to enhance the reproducibility of your results. Protocols.io assigns your protocol its own identifier (DOI) so that it can be cited independently in the future. For instructions see: https://journals.plos.org/plosone/s/submission-guidelines#loc-laboratory-protocols. Additionally, PLOS ONE offers an option for publishing peer-reviewed Lab Protocol articles, which describe protocols hosted on protocols.io. Read more information on sharing protocols at https://plos.org/protocols?utm_medium=editorial-email&utm_source=authorletters&utm_campaign=protocols.

We look forward to receiving your revised manuscript.

Kind regards,

Mohammed H. Karrar Alsharif, Ph.D.

Academic Editor

PLOS ONE

Journal Requirements:

When submitting your revision, we need you to address these additional requirements.

1. Please ensure that your manuscript meets PLOS ONE's style requirements, including those for file naming. The PLOS ONE style templates can be found at

https://journals.plos.org/plosone/s/file?id=wjVg/PLOSOne_formatting_sample_main_body.pdf and

https://journals.plos.org/plosone/s/file?id=ba62/PLOSOne_formatting_sample_title_authors_affiliations.pdf

2. In your Data Availability statement, you have not specified where the minimal data set underlying the results described in your manuscript can be found. PLOS defines a study's minimal data set as the underlying data used to reach the conclusions drawn in the manuscript and any additional data required to replicate the reported study findings in their entirety. All PLOS journals require that the minimal data set be made fully available. For more information about our data policy, please see http://journals.plos.org/plosone/s/data-availability.

Upon re-submitting your revised manuscript, please upload your study’s minimal underlying data set as either Supporting Information files or to a stable, public repository and include the relevant URLs, DOIs, or accession numbers within your revised cover letter. For a list of acceptable repositories, please see http://journals.plos.org/plosone/s/data-availability#loc-recommended-repositories. Any potentially identifying patient information must be fully anonymized.

Important: If there are ethical or legal restrictions to sharing your data publicly, please explain these restrictions in detail. Please see our guidelines for more information on what we consider unacceptable restrictions to publicly sharing data: http://journals.plos.org/plosone/s/data-availability#loc-unacceptable-data-access-restrictions. Note that it is not acceptable for the authors to be the sole named individuals responsible for ensuring data access.

We will update your Data Availability statement to reflect the information you provide in your cover letter.

3. Please include captions for your Supporting Information files at the end of your manuscript, and update any in-text citations to match accordingly. Please see our Supporting Information guidelines for more information: http://journals.plos.org/plosone/s/supporting-information.

4. Please review your reference list to ensure that it is complete and correct. If you have cited papers that have been retracted, please include the rationale for doing so in the manuscript text, or remove these references and replace them with relevant current references. Any changes to the reference list should be mentioned in the rebuttal letter that accompanies your revised manuscript. If you need to cite a retracted article, indicate the article’s retracted status in the References list and also include a citation and full reference for the retraction notice.

[Note: HTML markup is below. Please do not edit.]

Reviewers' comments:

Reviewer's Responses to Questions

**Comments to the Author**

1. Is the manuscript technically sound, and do the data support the conclusions?

The manuscript must describe a technically sound piece of scientific research with data that supports the conclusions. Experiments must have been conducted rigorously, with appropriate controls, replication, and sample sizes. The conclusions must be drawn appropriately based on the data presented. 

Reviewer #1: Yes

Reviewer #2: Partly

**********

2. Has the statistical analysis been performed appropriately and rigorously? 

Reviewer #1: I Don't Know

Reviewer #2: Yes

**********

3. Have the authors made all data underlying the findings in their manuscript fully available?

The PLOS Data policy requires authors to make all data underlying the findings described in their manuscript fully available without restriction, with rare exception (please refer to the Data Availability Statement in the manuscript PDF file). The data should be provided as part of the manuscript or its supporting information, or deposited to a public repository. For example, in addition to summary statistics, the data points behind means, medians and variance measures should be available. If there are restrictions on publicly sharing data—e.g. participant privacy or use of data from a third party—those must be specified.

Reviewer #1: No

Reviewer #2: Yes

**********

4. Is the manuscript presented in an intelligible fashion and written in standard English?

PLOS ONE does not copyedit accepted manuscripts, so the language in submitted articles must be clear, correct, and unambiguous. Any typographical or grammatical errors should be corrected at revision, so please note any specific errors here.

Reviewer #1: Yes

Reviewer #2: Yes

**********

5. Review Comments to the Author

Please use the space provided to explain your answers to the questions above. You may also include additional comments for the author, including concerns about dual publication, research ethics, or publication ethics. (Please upload your review as an attachment if it exceeds 20,000 characters)

Reviewer #1: This study by alqarawi and colleagues explores sudden death in Saudi, especially in relation to autopsy and family history. Overall, the knowledge on SD is very low in Saudi.

The study is mainly surveyed based distributed through online social media platforms. Participants’ characteristics, details about SUD cases, and the attitude toward medical autopsy and family screening were collected.

Main results are that the prevalence of FHx of at least one first degree relative (FDR) with SUD was found to be 9.4%. Among participants with any FHx of SUD, about one-third had > 2 family members affected. Only rarely did this result in contact with health care system. Interestingly only one in five had a negative attitude towards autopsy.

Even though the study have major limitations in its design, this study represents very important data and new knowledge deriving from a part of the world that do not represent the data we usually refer to when discussing SCD. The authors are to be commended for this!

I would moderate the conclusion in abstract. You state “SUD occurred at young age and affected multiple family members in a significant proportion of families. Despite that, family screening was seldom performed.” This is not wrong, but you have to keep in mind, that the sample you are describing do not represent the general population in Saudi. This is a Social Media questionnaire that only reached some parts of the community (the young?), and maybe only were returned by those severely affected by the questions? Nonetheless, the data are very disturbing and should warrant further investigations and information campaigns et cetera.

Limitations:

Bias on collection through SoMe of course makes it impossible to say that this cohort represents the general population. They are most likely younger and more urban?

Who answers these questionnaires? And more important – who wont receive the questionnaire?

This should be discussed too

Line 216-218: The yield in family screening in families with structural heart disease is higher, and in SADS (negative autopsy) around 20%. The yield is lower in non-autopsied families, presumably because not all SD are in fact SCD (and not all SCD are due to inherited cardiac disease). Please revise

On a smaller note, I would keep SUD for those that are unexplained, either because no autopsy was performed or because autopsy did not reveal a cause. I would use the term SD (sudden death) as a collection of these entities, which could then be divided in non-autopsied SD (which could then be SUD or SCD pr definition), and autopsied SD, which could then be non-cardiac SD, SCD or SUD/SADS. This would also be in line with current guidelines

I think this paper is very important, politically it should be used to affect policy makers, and to design campaigns towards the population to raise awareness on this important topic

Reviewer #2: Saudi within the title and manuscript should be replace by Saudi Arabia.

I believe the title should be a bit edited by deleting the number of participants and adding independent predictors of negative attitude toward medical autopsy. therefore, I suggest the title to be: Prevalence, Characteristics of Family History of Sudden Unexplained Death and Predictors of Negative Attitude of Family Members Toward Medical Autopsy and Family Screening in Saudi Arabia: A Cross-Sectional Study.

The objective should be strictly aligned with the title. I do not see prevalence in the objective statement.

- FHx should be defined.

-"The Saudi population has a unique age distribution and religious and cultural practices that might affect the prevalence and causes of SUD and care provided to their families". This sentence should be rewritten to add clarity and avoid any confusion in regard of age and practices with causes and care.

-Details about questionnaire development, validation and reliability should be provided in the methods section.

_ the prevalence was not clearly given, I believe it is 5474? or 3489?!. I believe a better presentation of outputs statring from larger to smaller should be presented clearly.

limit geographical location to cities rather than cities and regions( Eastern and Northern to be replaced by corresponding cities)

**********

6. PLOS authors have the option to publish the peer review history of their article (what does this mean?). If published, this will include your full peer review and any attached files.

If you choose “no”, your identity will remain anonymous but your review may still be made public.

**Do you want your identity to be public for this peer review?** For information about this choice, including consent withdrawal, please see our Privacy Policy.

Reviewer #1: No

Reviewer #2: **Yes: **Mohamad Al Tannir

**********

[NOTE: If reviewer comments were submitted as an attachment file, they will be attached to this email and accessible via the submission site. Please log into your account, locate the manuscript record, and check for the action link "View Attachments". If this link does not appear, there are no attachment files.]

While revising your submission, please upload your figure files to the Preflight Analysis and Conversion Engine (PACE) digital diagnostic tool, https://pacev2.apexcovantage.com/. PACE helps ensure that figures meet PLOS requirements. To use PACE, you must first register as a user. Registration is free. Then, login and navigate to the UPLOAD tab, where you will find detailed instructions on how to use the tool. If you encounter any issues or have any questions when using PACE, please email PLOS at figures@plos.org. Please note that Supporting Information files do not need this step.

# Author comment: Author response to Decision Letter 0

**DOI:** 10.1371/journal.pone.0277914.r002

4 Nov 2022

Dear Dr. Chenette,

The authors would like to thank the editor and reviewers for taking the time to appraise our manuscript. We have revised the manuscript based on the reviewers’ suggestions. The comments are in bold and our responses follow. 

Reviewer #1

Even though the study have major limitations in its design, this study represents very important data and new knowledge deriving from a part of the world that do not represent the data we usually refer to when discussing SCD. The authors are to be commended for this!

Thank you. Indeed, we hope that this manuscript will raise awareness and inform decisions about the care of SCD not only in Saudi Arabia but also in other countries that share similar cultural and religious backgrounds. 

I would moderate the conclusion in abstract. You state “SUD occurred at young age and affected multiple family members in a significant proportion of families. Despite that, family screening was seldom performed.” This is not wrong, but you have to keep in mind, that the sample you are describing do not represent the general population in Saudi. This is a Social Media questionnaire that only reached some parts of the community (the young?), and maybe only were returned by those severely affected by the questions? Nonetheless, the data are very disturbing and should warrant further investigations and information campaigns et cetera

Limitations:

Bias on collection through SoMe of course makes it impossible to say that this cohort represents the general population. They are most likely younger and more urban?

Who answers these questionnaires? And more important – who wont receive the questionnaire?

This should be discussed too

Thank you for allowing us to clarify this important point. We completely agree with the reviewer that selection bias is an important limitation of our study which might have affected the characteristics of responders. We have revised our conclusion in the abstract and revised our limitations to discuss selection bias.

• Line 62: Revised “SUD occurred at young age and affected multiple family members in a significant proportion of responders”

• Line 241: Added “There is certainly a selection bias when using social medial platforms where certain populations are less likely to be reached such as older individuals, rural populations and those with low socioeconomic status.” 

Line 216-218: The yield in family screening in families with structural heart disease is higher, and in SADS (negative autopsy) around 20%. The yield is lower in non-autopsied families, presumably because not all SD are in fact SCD (and not all SCD are due to inherited cardiac disease). Please revise

Thank you for your thoughtful comment. We have revised the manuscript.

• Line 218: Revised “As such, it is reasonable to postulate that the yield might be different when autopsy is systematically not performed (like the practice in Saudi), and one needs to study this in order to define the yield of family screening.

On a smaller note, I would keep SUD for those that are unexplained, either because no autopsy was performed or because autopsy did not reveal a cause. I would use the term SD (sudden death) as a collection of these entities, which could then be divided in non-autopsied SD (which could then be SUD or SCD pr definition), and autopsied SD, which could then be non-cardiac SD, SCD or SUD/SADS. This would also be in line with current guidelines

I think this paper is very important, politically it should be used to affect policy makers, and to design campaigns towards the population to raise awareness on this important topic

We have revised our manuscript to clarify the definition of SUD, as suggested.

• Line 70: Added “(either because no autopsy was performed or because autopsy did not reveal a cause)”. 

Reviewer #2

Saudi within the title and manuscript should be replace by Saudi Arabia.

“Saudi” was replaced by “Saudi Arabia” throughout the manuscript.

I believe the title should be a bit edited by deleting the number of participants and adding independent predictors of negative attitude toward medical autopsy. therefore, I suggest the title to be: Prevalence, Characteristics of Family History of Sudden Unexplained Death and Predictors of Negative Attitude of Family Members Toward Medical Autopsy and Family Screening in Saudi Arabia: A Cross-Sectional Study.

We have revised both the long and short titles as suggested.

• Line 1: Revised “Prevalence and Characteristics of Family History of Sudden Unexplained Death and Predictors of Negative Attitude of Family Members Toward Medical Autopsy and Family Screening in Saudi Arabia: A Cross-Sectional Study.”

• Line 6: Revised “Family History of Sudden Unexplained Death in Saudi Arabia: A Cross-Sectional Study”

The objective should be strictly aligned with the title. I do not see prevalence in the objective statement.

Thank you for highlighting this important point. We have revised the manuscript to add this objective.

• Line 42: Added “We sought to describe the prevalence and characteristics of family history of SUD and the attitude of family members toward medical autopsy and family screening”.

• Line 81: Added “As such, we conducted this study to describe the prevalence and characteristics of SUD, the care provided to family members and their attitudes toward screening and medical autopsy.”

FHx should be defined.

Manuscript was revised to define FHx.

• Line 89: Added “and family history of SUD was defined as having any first or second degree relative who had SUD.”

"The Saudi population has a unique age distribution and religious and cultural practices that might affect the prevalence and causes of SUD and care provided to their families". This sentence should be rewritten to add clarity and avoid any confusion in regard of age and practices with causes and care.

The sentence was revised.

• Line 79: Revised “The Saudi population has a unique age distribution that might affect the prevalence and causes of SUD and religious and cultural practices that influence the care provided to families with a history of SUD.”

Details about questionnaire development, validation and reliability should be provided in the methods section.

Thank you for your suggestion. We added a sub-heading in the method section detailing our questionnaire’s development and content and discussed the assessment of validity and reliability of the questionnaire. We also added to our discussion a limitation of not calculating any formal statistics related to reliability and validity, although we contacted responders during the pilot phase to ensure these qualities. 

• Line 86: Added a subheading “Questionnaire development and content”

• Line 259: Added “Third, although we contacted responders to ensure reliability and validity of the questionnaire, we did not calculate any formal statistics to objectively report that.”

the prevalence was not clearly given, I believe it is 5474? or 3489?!. I believe a better presentation of outputs statring from larger to smaller should be presented clearly.

Thank you for allowing us to clarify this important point. The number of participants with a family history of SUD was 3489 (3489/11374, 30.7%). However, some of them had more than one family member with SUD and, as such, the total number of SUD cases was higher (5474 SUD cases). We have updated our manuscript with a figure that explains these numbers. 

• Added the following figure (Fig 1):

Fig 1: Study flowchart depicting the prevalence of family history of sudden unexplained death and the number of sudden explain death cases. FHx: family history, SUD: sudden unexplained death, SUD-FDR: sudden unexplained death in at least one first degree relative, SUD-SDR: sudden unexplained death in second degree relative(s).

limit geographical location to cities rather than cities and regions( Eastern and Northern to be replaced by corresponding cities)

All geographical locations are regions. Some regions are named after the main city (e.g. Riyadh region, Mecca region) and others were named according to their location (e.g. Eastern region, Northern region). We added the word “region” to their names to avoid any confusion

• Table 1. Added “region” to all regions.

Attachment Submitted filename: Response to Reviewers.docx Click here for additional data file.

# Editor report: Decision Letter 1

**DOI:** 10.1371/journal.pone.0277914.r003

**Authors:** Mohammed H. Karrar Alsharif

6 Nov 2022

Prevalence and Characteristics of Family History of Sudden Unexplained Death and Predictors of Negative Attitude of Family Members Toward Medical Autopsy and Family Screening in Saudi Arabia: A Cross-Sectional Study.

PONE-D-22-22713R1

Dear Dr. alqarawi,

We’re pleased to inform you that your manuscript has been judged scientifically suitable for publication and will be formally accepted for publication once it meets all outstanding technical requirements.

Within one week, you’ll receive an e-mail detailing the required amendments. When these have been addressed, you’ll receive a formal acceptance letter and your manuscript will be scheduled for publication.

An invoice for payment will follow shortly after the formal acceptance. To ensure an efficient process, please log into Editorial Manager at http://www.editorialmanager.com/pone/, click the 'Update My Information' link at the top of the page, and double check that your user information is up-to-date. If you have any billing related questions, please contact our Author Billing department directly at authorbilling@plos.org.

If your institution or institutions have a press office, please notify them about your upcoming paper to help maximize its impact. If they’ll be preparing press materials, please inform our press team as soon as possible -- no later than 48 hours after receiving the formal acceptance. Your manuscript will remain under strict press embargo until 2 pm Eastern Time on the date of publication. For more information, please contact onepress@plos.org.

Kind regards,

Dr. Mohammed H. Karrar Alsharif, Ph.D.

Academic Editor

PLOS ONE

Additional Editor Comments (optional):

Reviewers' comments:

# Editor report: Acceptance letter

**DOI:** 10.1371/journal.pone.0277914.r004

**Authors:** Mohammed H. Karrar Alsharif

14 Nov 2022

PONE-D-22-22713R1 

Prevalence and Characteristics of Family History of Sudden Unexplained Death and Predictors of Negative Attitude of Family Members Toward Medical Autopsy and Family Screening in Saudi Arabia: A Cross-Sectional Study. 

Dear Dr. Alqarawi:

I'm pleased to inform you that your manuscript has been deemed suitable for publication in PLOS ONE. Congratulations! Your manuscript is now with our production department. 

If your institution or institutions have a press office, please let them know about your upcoming paper now to help maximize its impact. If they'll be preparing press materials, please inform our press team within the next 48 hours. Your manuscript will remain under strict press embargo until 2 pm Eastern Time on the date of publication. For more information please contact onepress@plos.org.

If we can help with anything else, please email us at plosone@plos.org. 

Thank you for submitting your work to PLOS ONE and supporting open access. 

Kind regards, 

PLOS ONE Editorial Office Staff

on behalf of

Dr. Mohammed H. Karrar Alsharif 

Academic Editor

PLOS ONE
